# Maintaining Factors of Anorexia Nervosa Addressed from a Psychotherapeutic Group for Parents: Supplementary Report of a Patient’s Therapeutic Success

**DOI:** 10.3390/ijerph191811396

**Published:** 2022-09-10

**Authors:** María García-Anaya, Alejandro Caballero-Romo, Laura González-Macías

**Affiliations:** 1 Clinical Research Division, National Institute of Psychiatry Ramón de la Fuente Muñiz, Mexico City 14370, Mexico; 2 Eating Disorders Clinic, Clinical Services Division, National Institute of Psychiatry Ramón de la Fuente Muñiz, Mexico City 14370, Mexico

**Keywords:** anorexia nervosa, reflective function, maintaining factors, psychotherapeutic group for parents, vicarious learning, intergenerational transmission

## Abstract

(1) Background: Anorexia nervosa is an eating disorder (ED) where up to 30% of individuals remain unresponsive to treatments, whether they partially respond, or do respond and later relapse. It has been broadly reported how presenting maladaptive family functioning and communication style contributes to treatment drop-out, poor treatment compliance, and poor long-term outcomes. We studied the mother and father of a patient with AN, binge-purge subtype (according to DSM-IV TR) who achieved remission after her parents but not her attended an intervention through a psychotherapy group for parents (PGP). (2) Methods: We previously reported this patient’s case report, and now, through an Interpretative Phenomenological Analysis (IPA) approach, we aimed to explore the understanding and meanings ascribed by the mother and father to their experience at the PGP and to their daughter’s clinical and functional improvement. (3) Results: We identified two main stages along the process: one related to the presence of maintaining factors of their daughter’s disorder, and the other related to the emergence of a reflective function and to the implementation of behavioral, emotional and cognitive changes. (4) Conclusions: The interview revealed both parents’ experience at the PGP promoted a change process, where they were able to modify their previous style of communication and functioning, and to identify them as a contributors to maintain their daughter’s disorder. Reflective function (RF) emerged in the mother and father throughout the psychotherapeutic process. Both parents also revealed some elements that were intergenerationally transmitted, that affected three generations and contributed to maintaining the ED. We observed the multilevel open-group structure of the PGP, enhancing the mother’s and father’s change process.

## 1. Introduction

Anorexia nervosa (AN) is an ED that affects up to 3.6% of females and 0.3% of males [1]. It is characterized by restriction of energy intake relative to requirements, intense fear of gaining weight or becoming fat, and disturbance in the way in which one’s body weight or shape is experienced. Over the years, it has been observed that the course and outcome of AN is highly variable. Even when most individuals succeed in resolving the ED, approximately 30% of them remain unresponsive; whether they partially respond, or they present good response to treatment but later they relapse [2]. Furthermore, it is worth mentioning how these individuals are very prone to refuse, avoid, or drop out of treatment [3]; three conditions that impair their prognosis and increases the risk of suffering serious medical consequences [4,5]. Therapeutic options for individuals with poor response to standard treatment, with an evolution tending to chronicity and for adults in general, are few and have not been shown to be as effective as for adolescents [2,6,7]. So given the high risk for severity and impairment of individual’s functioning that AN may cause [8,9,10], it is crucial to aim efforts at developing tailored therapeutic proposals for those cases with prior poor response to treatment. By saying tailored we refer to individualized assessment of characteristics [11,12] that could lead us to identify the particular needs of each case and to address them in that way. For example, at the Clinic of Eating Disorders (CED) of the National Institute of Psychiatry, Ramón de la Fuente Muñiz, in Mexico City, we have conducted for the last 20 years a PGP oriented to families (particularly parents) of patients with a history of poor response to treatment and tendency to chronification. Among them, we have observed how they share styles of communication and functioning corresponding to the ones described by classical [13,14,15,16,17] and contemporary authors [18,19,20]. Through the years, we have also observed how those styles contribute to maintain AN; so, we find in such styles, characteristics worth being individually assessed and addressed in order to improve therapeutic outcomes.

From the point of view of systemic therapy, communication in families sharing such styles of functioning and communicating, occurs through the ED, which becomes the necessary channel to connect and cope with the emotional world [15]. This mechanism has also been described as characteristic of alexithymic families [21]. Alexithymia (from the Greek roots: a- lack of; lexis- word; thymos- emotion) refers to the inability to identify, describe and express emotions [22,23]. So, in these families the emergence of clinical manifestations helps them avoid conflict and emotional stress through the somatic expression of symptoms [24]. The style of functioning of families has been operationalized under the construct of Expressed Emotion (EE) [25,26]. EE refers to the way families relate and communicate between them in terms of emotions, attitudes, and behaviors, expressed as high criticism, or emotional over-involvement by relatives about a family member diagnosed with a mental disorder [27,28,29,30,31,32,33,34]. We have also observed these families’ functioning matches with the elevated scores of expressed emotion that has been reported to contribute to treatment drop-out, poor treatment compliance, and poor long-term outcomes [35,36,37,38,39,40,41,42].

**Figure 1:**
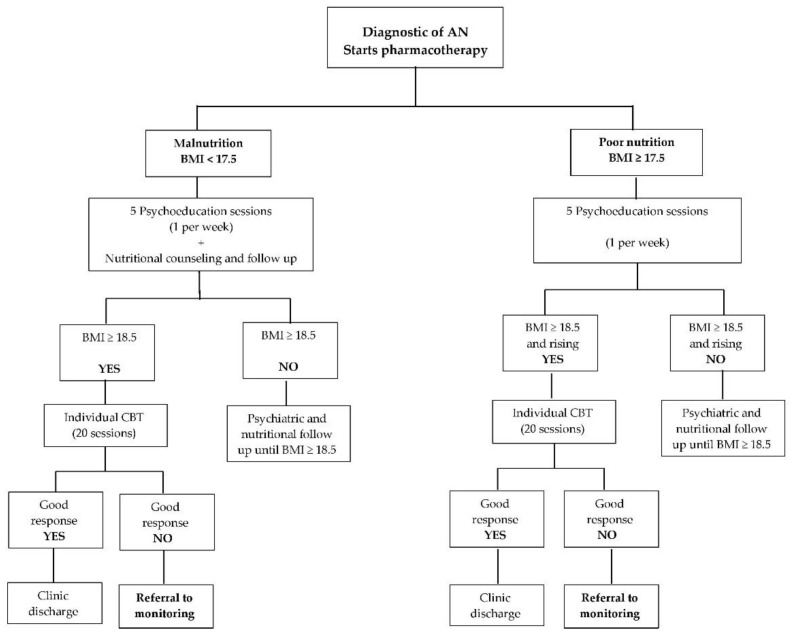
Algorithm of treatment for AN used at the CED. Time elapsed from admission to the clinic to referral to monitoring, varies depending on each patient’s individual response, number of relapses, and adherence to treatment.

We previously published an emblematic case report where we followed the clinical evolution of a patient with AN, binge-purge subtype (according to DSM-IV TR), whose family presented characteristics like the ones mentioned above. She received standard treatment (pharmacotherapy, psychoeducation, cognitive behavioral therapy [CBT], nutritional counseling) for enough time a dose according to clinical guidelines (Figure 1). She partially improved, showing decreasing of purging and compensatory behaviors, increasing of Body Mass Index (BMI) from 16.0 to 18.5, and decreasing of compulsive exercise; however, she remained at cognitive impasse for three years, which progressively impacted her global functioning leading her to relapse. Attention at the CED was resumed, so this time she was referred to the monitoring protocol intended to manage unresponsive cases, which contemplates: (1) Providing general support to patients; comprising pharmacotherapy, appointments with the psychiatrist every six to twelve weeks, and attendance to the psychotherapeutic group of patients at risk of chronification; (2) Working with the patient’s family through the PGP. The daughter and her parents started the monitoring protocol, however, after six weeks, the patient declined to continue treatment. Thus, she first dropped out of psychotherapy, and later (said by her parents) pharmacotherapy as well. Nevertheless, in the face of their daughter’s reluctance to accept treatment, we highly encouraged the mother and father to continue attending the PGP, and they did. Throughout the mother’s and father’s attendance to the group, daughter’s global functioning and family relationship started steadily improving. She abandoned purging and compensatory behaviors, reach a health body mass index, and voluntarily resumed pharmacotherapy [43].

The daughter’s evolution linked to the notion that certain elements present in family dynamics may be operating in the improvement or worsening of some patients [44,45], we hypothesized that maybe these mother and father and their family functioning could be somehow contributing to maintaining their daughter’s AN. Our hypothesis is supported in the clinical observation of the PGP for 20 years, and in the particular evolution of this emblematic case: improvement of the daughter’s global functioning and clinical condition after her mother and father attended the PGP. We also hypothesized that in case they were contributing to the maintenance of AN, such contribution could be related to difficulties reflecting on their own behaviors and functioning style. By “reflect” we refer to the psychological process mediated by RF, which “involves a self-reflective and an interpersonal component that provides the individual with the ability to distinguish inner from outer reality, pretend from ‘real’ modes of functioning, and from interpersonal communications” [46]. With the aim of understanding these aspects, we wanted to explore what happened regarding the mother’s and father’s experiences at the PGP, how they lived and signified it, and if that experience could be related to their daughter’s improvement.

## 2. Materials and Methods

We used a qualitative methodology to understand how the parents whose daughter declined to continue treatment while being in a severe relapse, understood and signified their process at the PGP. We chose to do IPA since it is committed to delve in the lived experience and in the meanings people ascribe to significant life events. Among qualitative researchers, it is considered the most participant-oriented approach [47].

A semi-structured interview was developed after conducting a study aimed at characterizing the intervention delivered at the PGP. We posed broad questions oriented to explore the parents’ experiences and understandings throughout their participation at the PGP. Questions were as follows: (1) Why did you seek treatment for your daughter? (2) What expectations did you have of your daughter’s treatment? (3) What do you understand about your daughter’s disorder? (4) How do you understand your daughter’s disorder? (5) How would you describe the PGP? (6) What experiences did you have in the PGP? (7) How was your process at the PGP? (8) What function did the PGP have for you? The responses emerging from these questions drew out elements that lead us to inquire about their origin families and about the way they perceive themselves as mother and father.

Interviews were recorded and transcribed. The father’s interview lasted 90 min, and the mother’s lasted 115 min.

Data analysis was made according to IPA procedures drawn up by Smith and cols [47]. From the transcripts, preliminary themes were identified from each interview and later grouped in related clusters. Themes were contrasted between the two interviews and then gathered into sub-themes. Afterwards, we made a participant check where both parents agreed with the coding. We additionally triangulated the emerging themes and sub-themes with data previously collected through observation sheets used to characterize the intervention at the PGP [48].

Both parents voluntarily agreed on conducting and recording the interview. They granted oral consent and also signed an informed consent.

Some quotes presented here disclose sensitive information related to parental behaviors and attitudes. The PGP sessions were conducted by 1 psychotherapist and 2 co-therapists with expertise to adequately address these comments using session containment, clarification, validation, and comforting among other therapeutic tools [48]. Parents were also referred to specialized attention to address these topics through individual and couple psychotherapy.

### Participant’s Description

Mother and Father: They are a married couple of middle socioeconomic status. Both of them completed a bachelor’s degree at university and they work as independent entrepreneurs. They were 48 (her) and 49 (him) years old at the time they started attending the PGP.

Daughter: She was 17 years old at the time she entered the CED. She was referred by the high school psychologist. Upon admission, she presented a BMI indicative of malnutrition (16.0), a frequency of vomiting of 70 per week, compulsive exercise, distortion of and dissatisfaction with her body image (she referred perceiving herself as “big and fat”), self-injuries, and risk behaviors such as casual sex and substance abuse. As for her global functioning she presented interpersonal troubles at home and school, poor academic performance, and defiant and violent behaviors towards her parents and other authority figures.

## 3. Results

We obtained the interviews three years after the daughter was discharged from the CED. Parents declared having attended for a period of three years.

Five themes and one sub-theme resulted from the analysis. The first four themes and sub-theme “ignorance about AN”, “my daughter’s behavior”, “realizing/epiphany”, “aware changes”, and “it had to do with me”, were interconnected through a consecutive-chronological relation where parents’ experiences referred to different stages in the process at the PGP where they observed, recognized, understood, and modified personal and family dynamics. The fifth theme “meaning of the group” had a cross-cutting relationship with the other themes and sub-theme. It was present throughout the experience as a supportive and reflective element.

### 3.1. Theme 1: Ignorance about AN (Maintainers Phase)

Parents described how the onset of AN was not obvious to them. They recognized they ignored what they were up to and what they should do. This recognition somehow seemed to entail it was not only a lack of knowledge of AN but something that could be interpreted as the need to maintain a status, or the resistance to change a status. This ignorance appeared to let them continue behaving as if they did not have a daughter with severe AN. They declared how even after having received a lot of information and instructions from the psychiatrist, psychotherapist, and nutritionist, and at the psychoeducational sessions, when they enter the PGP, they still ignored basic issues related to AN and their daughter’s treatment. The data suggest this ignorance lasted a long period of time and contributed to their being able to maintain behaviors that they later recognized had a negative impact on their daughter.

*“One hopes in two or three days they will give us the recipe and we will go home, I did not even think about weeks, much less months or years… After two suicide attempts, I was still in the same situation, and I attended and listened in the group, but I didn’t analyze, I didn´t make that analysis within the person”.*

(father)

*“I didn´t realize my daughter’s disorder, even the lady who worked for us did, but I didn´t… At first (when she started attending the PGP) I didn´t know what I was up to, we did not understand what the treatment consisted of, my expectation was that she would be cured with medication and she would oversee her nutritional plan, and I could go on with my work and my studies”.*

(mother)

### 3.2. Theme 2: My Daughter’s Behavior (Maintainers Phase)

Upon the entrance to the PGP, we have observed through the years that once novel parents start to open up to the experience of being in the group, their expressed concerns are regularly centered on their daughters’ behaviors. This is a particularly dense stage in the process of all parents, because on one hand, they repeatedly express the fear of losing their daughter, they talk about all troubles implementing treatment and getting their daughter to eat, and on the other, they remain focused on the way and amount of food their daughter eats, on the discussions around taking medication, the attitudes of their daughter towards them, and about their frustration for unsuccessful attempts to have their daughter cured. This stage could remind one of the experience of someone walking in circles; they identify how their daughter behaves and how they approach her the same way each time without results or changes, and they repeat it each session for long time. Data emerged from the interviews suggesting the mother and father´s insight into what is happening is kind of stuck in the experience of therapeutic failure.

*“At first one enters the group apathetic, ignorant, thinking that in two or three sessions they will give you the recipe and let’s go, we enter as victims, feeling attacked; at first one talks a lot about what our children do wrong, at first one has no focus but with the sessions one becomes participatory, one wants to learn… later, I used to see novel parents and think, ‘oh, what is left for them to go through’ but one helps them understand faster”*.

(father)

*“When I got to the group, I was very nervous, I wanted to run away... I wanted to go back to her childhood when she didn’t give me problems... At the beginning, we the parents of the group are angry, we are harsh control parents, overprotective, aggressive, stubborn, we have many expectations about our children, we complain about them all the time, that’s how I was, but over time that changes”.*

(mother)

### 3.3. Theme 3: Realizing/Epiphany (Emergence of RF Phase)

Along this theme, parents expressed that there came a moment in the process when they started noticing that besides their daughter´s behavior, there were other elements involved in the manifestation of AN. They recognized how they were also related to the disorder, and at this point both parents experienced how they turned to see themselves and their own behaviors. They realized they were somehow contributing to their daughter´s disorder. They appeared to experience those realizations as a consequence of their participation at the group. This information suggests what they realized seemed to open them up to the ability to see a new dimension related to their daughter´s disorder.

*“One day I realized I had some monsters, what monsters? Well, I was an absent father, an alcoholic father, an ambivalent father who didn´t set rules nor limits, there were no consequences for anything, not even to me, because I had gains such as comfort and freedom to go party, drink and hang out with other women… it hurts to realize that, but it helped me to see what I was like as a person”.*

(father)

*“At the group sessions I understood the things I did were to stop looking at her, and her behavior was telling me to love her, and to pay attention to her, but I didn’t understand... I understood I didn’t like her, and I had to admit when I was pregnant, I wanted a boy, not a girl.... I understood my participation in the ED when I made a table with the information, they gave us in the group. I wrote the profile of mother I was, and there I was. I understood I was harsh controlling, dominant, aggressive, overprotective, I recognized I used to check her backpack and rummaged through her drawers, and I kept her grounded for a very long periods, and I rejected her”.*

(mother)

### 3.4. Theme 4: Aware Changes (Consolidation of RF Phase)

The mother and father shared how their experience at the PGP fostered their ability to identify and change behaviors that were contributing to maintaining family dynamics they recognized negatively impacted the relation with their daughter and promoted the maintenance of her disorder. Data suggests the ability to observe themselves and modify what they realized, is a consequence of the emergence of RF.

*“I realized what I was doing but it was until I sat down and did a reflective exercise and thought about what my gains were with this situation, that I saw I was maintaining my daughter’s disorder. It was until that moment that I was able to change it… One has two paths, change, or turn a blind eye, so I made changes, I began to be a present father, a faithful husband, I began to set clear limits but with love, not violence”.*

(father)

*“The process in the group was trial and error, first I didn’t realize the role I had to play as a mother, then I understood I did things affecting my daughter’s emotions, but I didn’t know how to change it, and later I began to realize how to do it. Now, I am present without being controlling, now I set limits, but I give her freedom to make her decisions, now I contain her emotions and I give her responsibility back”.*

(mother)

### 3.5. Theme 4a: It Had to Do with Me (Consolidation of RF Phase)

In this sub-theme, both the mother and father talked about having recognized aspects of their own history that emerged along with the understanding of their daughter’s disorder and their contribution to maintaining it. They appear to find a link between what they were doing as parents, and how they functioned as son and daughter, or brother and sister. It seems the way their origin families functioned was somehow determining their current family´s functioning. They both realized their own responsibility and their will to change that way of functioning. Data suggest their realizations entail the repetition and intergenerational transmission of family-learnt behavioral patterns.

*“When I realized what I was like, I wanted to die, I felt like the worst dad, and let’s not talk about what I was like as a husband. I realized how I was as a person, I understood that I also had my story as a son, as a brother and I had to take care of it, working on myself”.*

(father)

*“The group was a training to be a better mother, partner, daughter, aunt. I was able to cut the bond with a mother as difficult as the one I have; I will not allow her to treat me badly again. Many years I neglected my daughters to keep her happy”.*

(mother)

### 3.6. Theme 5: Meaning of the Group (Vicarious Learning)

One of the main features of the PGP is that it holds a multilevel scheme where novel and veteran parents co-participate and contribute to each other process. The group seems to constitute a place where the mother and father went to seek for help and advice, where they received information related to their daughter´s disorder, where they spoke out the way they felt and thought, where they received contention, comfort, and support, where they mirrored in each other, where they shared their experiences, and learnt from each other. It seems the group played the roll expected for a therapeutic group in terms of listening, containing, informing, supporting, and identifying, however it appears the group also provided an experience not necessarily attributable to every therapeutic group; it is vicarious learning. Data suggests the changes experienced by the mother and father, do not only stem from the psychotherapist’s intervention but by the vicarious experience at the group. It appears vicarious experience enhanced parent´s learning.

*“I called it mirror therapy, one speaks and is mirrored in the other, and thus we see the past, present, and future of the families that are there… I saw my future in a veteran couple, they were my reference, I heard them and said—what they are telling is my past, and I want that future… I experienced moments of pain, sadness, guilt, anger; many emotions, there I got undressed… I used to mirror myself on other parents, one says, –‘oh, that is me talking and I look ridiculous, I look bad, I look out of place, what a shame’, however I felt there is a contention. The parents hit us, but then give us a little kneading so you can go quietly to think about things... for me it was as if they opened my mind and understanding”.*

(father)

*“The group has a very important feedback process. It was a place where I felt very sheltered, and learnt from other parents, the group gave me a lot of love and they recognized the work I did … The group serves to train us to be mothers and fathers, to be couples, to be better persons... my daughter is now functional, and the group was the vehicle to get to this point”.*

(mother)

## 4. Discussion

Through the mother’s and father’s narratives we could observe how their experience at the PGP fostered a complex sequence of profound personal changes. The themes resulting from the analysis, led us down the path they traveled through various phases. We identified two main stages along the process. The pre-reflective stage refers to the period of time where they presented a superficial recognition of behaviors, emotions, and cognitions related to the phenomenon. This stage involves elements we consider as maintaining factors of the disorder, and it included the themes “ignorance of AN”, and “my daughter’s behavior”. The reflective stage, refers to the period of time where they were able to recognize the behaviors they had to observe and from that, they reflected about themselves. This stage included the themes “realizing/epiphany”, “aware changes”, and the sub-theme “it had to do with me”.

At the beginning of the process, while speaking of “ignorance of AN”, we could see how the experience of the mother and father was of denial of their daughter’s disorder. It draws attention to how even when they themselves had coped next to her, and had attended several psychoeducational sessions, and medical, nutritional, and psychotherapeutic appointments for long time, they kept believing a pill and attendance to two or three sessions at the PGP could be enough to have their daughter cured. When they spoke about “my daughter’s behavior” we could see the transition to start observing something being there for years, but they were not able to see. Here, even when they now were speaking about what their daughter was doing wrong and they were asking for advice and help, we find denial was still implied in the notion that they had nothing to do to help improve their daughter’s condition beyond getting her to accomplish pharmacotherapy and her dietary plan. We point to denial since they had previously centered their efforts exactly on that task, getting her to eat and take medication, with poor results (improved BMI and decreased purging behaviors and compulsive exercise, but global functioning was progressively worsening) [43]. Data indicate they were not observing their previous experience, they were not considering implementing new strategies, they were not reflecting on aspects other than persuading her to eat and take medication. At the PGP, all parents are continuously invited to observe and reflect on the way they act, feel, and think in relation to her daughter´s behavior. We could observe it takes some time for them to be able to do it [48], so we believe at this point this mother and father were stuck on the experience of treatment failure. The mother’s and father’s narratives show at this stage their ability to perform an intra-personal mental and emotional process (meaning to exert their RF) is reduced, so they recurrently return to focus on their daughter’s behavior to resolve the situation rather than reflecting over themselves. It has been argued before, how maternal/parental ability to contain, mirror, and handle the child’s intense and difficult emotional expressions (emotion regulation), enhances the child’s ability to effectively regulate emotions, which consequently maintain attachment and mentalization systems activated [49,50,51]. This description refers to an attribute that, at this stage of the process, is not present in the mother and father.

The mother’s and father’s narratives related to the theme “realizing/epiphany” let us see the psychotherapeutic work began to yield results and they started being receptive to listening and observing themselves. This ability to self-reflect and distinguish between inner and outer reality, meaning RF, was there operating in the way they perceived their daughter’s condition and how they related to it. This effect seems to be due to two main interventions, where on one hand co-participants were crucial for them to realize their own behaviors and thoughts through situations shared and mirrored among all parents. On the other hand, the psychotherapist’s intervention played a central role in fostering reflection and understanding. It worth mentioning that even when it did not constitute a theme itself, during the interview both mother and father, expressed they recognized the psychotherapist as a metabolizer of the information shared at the group, so they were able to understand and identify it. Regarding this, we present:

*“At the group there is a lot of information and the psychotherapist, mmh, it is as if she had a blender, she blends everything and returns it already digested, ready to understand it, so we can learn from what is happening”.*

(father)

*“I will never be grateful enough to the psychotherapist for helping us. It was thanks to her experience and her work that I was able to see myself. It must be very tiring to do her job, pointing out and patiently explaining the same things over and over again for years, until she gets us to see ourselves”.*

(mother)

What we understand up to here is that the psychotherapist played a foster–caregiver role, symbolically offering the mother and father an environment of emotional containment needed to promote the ability to mentalize, especially when negative and ambivalent mental states were shared at the group sessions [49]. The multilevel scheme of the group participants seemed to potentiate the development of RF through a vicarious learning mechanism [52], where the mother and father mirrored on each other from the experiences shared at the group sessions, and they learnt new patterns of behavior from the observation of other parents [53], particularly from the veteran ones.

When the mother and father spoke about “aware changes” they let us see how their ability to listen and observe themselves had been strengthened and they were able to draw on their RF to identify how they had been behaving, feeling, and thinking, and to recognize the functioning style they held. We know EE has shown to be a reliable predictor of relapse of many psychiatric disorders, including AN [27], and a predictor of treatment drop-out and poor treatment compliance in patients with AN [35,36,37,38,39,40,41,42]. Data showed how the mother and father increased RF, enabled the understanding of the link between their communication and functioning style, and the negative behavioral characteristics that facilitated the maintenance of their daughter´s disorder. Furthermore, they were able to figure out their own strategies to modify their functioning style into more adaptative ways to relate at the personal and family level.

Narratives associated to sub-theme “it had to do with me” exposed some personal features of the mother and father, related to the level of participation they acknowledged their origin families had in the way they functioned and behaved. Eating behavior is determined by biological, psychological, and social [54] components, and over the individual suffering the disorder there are always operating genetic and environmental conditions that precipitate and maintain the disorder. When above in this text we pointed to the mother and father having a role in the maintenance of the disorder, we are referring to the effect of behavioral elements observable through the style of communication and functioning they hold with their daughter and their family, the current and the origin one.

After 20 years conducting the PGP, we have been able to consistently observe throughout the generations how the style of communication and functioning, and even some personal features present in the mother and father studied in this article, are recurrently present in other parents attending the PGP. By saying this, we postulate such characteristics observed through the analysis we conducted here, are somehow generalizable to other parents. Among the mentioned maintaining factors we cannot ignore the fact that the same genetic components exist for patients and for parents, who in turn have the same predispositions in the sense of how each member of the family will be determined by their own family’s trajectory [55,56,57,58,59,60,61,62,63,64], which leads us to less determined variables that could be participating in the predisposition, precipitation, and perpetuation of AN. By saying this, we do not mean in any way to blame parents for their children’s disorder, nor to find the reason why the disorder occurs in families holding this style of functioning. Rather, we seek to understand how parents have functioned at their current family and in their origin families as well; what we believe represent an important antecedent that conditions their style of functioning. We acknowledge parents do their best from their own personal resources and understanding of situations, but we cannot ignore that each one of them has their own story to tell and their own complex functioning system, which must be combined with their partner’s functioning system to direct them towards the same goal, raising a child. We also must consider each parent has their own cultural baggage, acting as a determinant element throughout their life, and possibly, during their daughter’s disorder, where chances for disagreement and dysfunction become evident.

We identified that from the intervention at the PGP, the mother and father achieved the observation of (1) their own functioning style, as well as that of their families of origin, (2) their learnt style of communication, (3) their own disordered relationship with food [43], and (4) the dysfunctional relationship with their own parents. All these show us a trigenerational condition, as far as we can trace it. This sub-theme presented a condition where family functioning style, RF, and personal history of parents converges as three factors related to their daughter’s AN. We found the mother’s and father’s narrative suggests a relation among these factors. To our knowledge, there are few reports that have directly studied how parents’ personal history contributes in terms of parenting risks to their child’s outcomes [65,66]. In contrast, family functioning style [35,36,37,38,39,40,41,42] and RF [67,68,69,70,71] have been well studied as factors impacting the evolution and outcome of AN. However, we believe the relation between these three factors is poorly studied and understood. We understand the difficulty involved in studying these three factors, however, were not able to locate a single study assessing/documenting such interrelation. We do believe the information emerging from this sub-theme opened a new dimension related to therapeutic potential alternatives to develop individualized interventions to address certain cases of individuals with AN with poor responses to treatment and a tendency to chronification. In other words, we mean we find in addressing of trigenerational elements implicated in the maintenance of AN, a therapeutic approach that is worth studying.

In regard to the last theme, “meaning of the group”, we found this notion was present throughout the whole narrative of the mother and father experience´s at the PGP. Our analysis let us see the group constituted a context and a complex instance that allowed, guided, contained, and enhanced their change process. We also could observe how it operated at cognitive, affective, experiential, and even transcendental levels. Even when the PGP shares some characteristics with other psychotherapeutic groups [72,73,74,75], we hypothesized the good results we observed are given by certain elements we now describe. (1) The multilevel open scheme held by the group structure enabled vicarious learning from parent to parent through the transmission of personal experiences, especially from veterans to novel participants. It is important to mention that such transmission occurs in a horizontal context where parents are able to point out sensible information of their peers supported by their own experience, which allows the information to be received in a smooth manner. So, this becomes a self-revelation experience, where afterwards reflection may take place. It is worth saying that veteran parents have internalized the reflective style of the psychotherapist and the transference of information is somehow metabolized, as the information that otherwise would be provided by the psychotherapist. This condition contrasts with the interventions the psychotherapist does make, that occurs in a vertical manner and sometimes could be received by novel parents with skepticism. (2) The homogenous characteristics of the parents attending the PGP, in terms of their style of functioning and communicating, increases the chances of better response to this therapeutic intervention. (3) Given the psychodynamic frame of this intervention, the psychotherapeutic work is focused on promoting a deep reflection towards structural modifications, rather than behavioral changes.

Finally, through the analysis of the mother’s and father’s narratives, we were able to see how parents lived the experience at the PGP as a process of change where, on the one hand, RF emerged and, on the other, their functioning style became evident, which was contributing to maintaining the disorder. We could understand this style of functioning has an important relationship with the way they learnt at their family of origin about parenting. We found in trigenerational elements [55,56,57,58,59,60,61,62,63,64], the opportunity to address therapeutic efforts, when families holding the functioning styles described throughout this article are identified. From these findings, we believe it is needed to refine screening instruments to identify this type of family. We acknowledge this style of functioning is not present in all families of individuals with AN with a history of poor response to treatment or with a tendency to chronification. However, we believe carrying out individualized evaluations to identify the cases in which these aspects are present, would contribute to address them and prevent chronification.

## 5. Conclusions

We were able to track the mother’s and father’s experiences at the PGP. We observed that the group fostered a complex sequence of profound personal changes in them. We identified two main stages along their change process, pre-reflective, related to the presence of maintaining factors of the disorder, and reflective, related to the emergence of RF. We were able to observe the emergence of RF when they progressed from one stage to another. Both the mother and father were able to identify the communication and functioning style they held in relation to their daughter and family, and they could also recognize how their style contributed to maintain their daughter’s disorder. The mother and father linked the presence of these factors to trigenerational elements that were contributing to maintain the disorder through intergenerational transmission. They also were able to modify their functioning style. Intergenerational transmission of functioning style and trigenerational elements, open a beta of research to broaden the understanding of chronification factors and to explore therapeutic alternatives for AN. Finally, the multilevel open group structure of the PGP is the distinctive feature of this psychotherapeutic intervention, which is an important enhancer in the process of emergence of RF through vicarious experience among participant parents.

## 6. Limitations

We acknowledge the use of a qualitative research method, particularly through IPA analysis represents a limitation since our observations are not generalizable. Instead, we offered a close insight into underlying elements implicated in notions such as RF, style of functioning, and intergenerational transmission of communication and functioning styles. We believe the results we present here will contribute to the construction of new evidence that allows generating valuable and reliable knowledge.

## Data Availability

The data presented in this study are available on request from the corresponding author. The data was obtained from in-depth recorded interviews with participants and are not publicly available due to privacy reasons.
